# Effect of hyperuricemia and treatment for hyperuricemia in Japanese hemodialysis patients: A cohort study

**DOI:** 10.1371/journal.pone.0217859

**Published:** 2019-06-06

**Authors:** Naoki Sugano, Yukio Maruyama, Satoshi Kidoguchi, Iwao Ohno, Atsushi Wada, Takashi Shigematsu, Ikuto Masakane, Takashi Yokoo

**Affiliations:** 1 Division of Nephrology and Hypertension, Department of Internal Medicine, The Jikei University School of Medicine, Tokyo, Japan; 2 Division of General Medicine, Department of Internal Medicine, The Jikei University School of Medicine, Tokyo, Japan; 3 Committee of Renal Data Registry, Japanese Society for Dialysis Therapy, Tokyo, Japan; International University of Health and Welfare, School of Medicine, JAPAN

## Abstract

Whether higher serum uric acid (UA) values comprise a risk factor for death and whether treatment for high UA is effective in patients undergoing hemodialysis (HD) are essentially unknown. To determine associations between UA and all-cause or cardiovascular (CV) mortality, interactions between UA or medication and effects on mortality, and significance of treatment for hyperuricemia in patients undergoing hemodialysis (HD). We collected the baseline data of 222,434 patients undergoing three HD sessions per week, extracted from a nationwide dialysis registry at the end of 2011 in Japan. Then we evaluated the interaction between serum uric acid level and all-cause and cardiovascular (CV) mortality by the end of 2012. Univariate and multivariate logistic regression and Cox regression analyses found higher all-cause and CV mortality rates among patients with lower, than higher UA values. Hazard ratios (HR) for all-cause and CV mortality were significantly lower in a group with, than without medication for hyperuricemia (HR, 0.837; 95% confidence interval (CI), 0.789–0.889 and HR, 0.830; 95%CI 0.758–0.909, respectively). Lower UA values remained associated with all-cause and CV mortality rates even when in patients taking medication for hyperuricemia. The chief interacting factors for higher mortality rates due to lower UA were higher BMI and diabetes mellitus. In conclusion, lower UA levels were independently associated with higher all-cause and CV mortality among Japanese patients undergoing HD. Intervention for hyperuricemia is considered to improve patient outcomes.

## Introduction

High serum uric acid (UA) values confer risk for gout and kidney damage and comprise a risk factor for cardiovascular (CV) events [1–6] among patients with normal renal function. Although a major antioxidant, UA is involved in hypertension, obesity, kidney and CV diseases, all of which are associated with oxidative stress [7–9]. Several studies have uncovered associations between UA values and all-cause and CV mortality among patients with chronic kidney disease (CKD) who do not undergo dialysis. Higher UA values are associated with higher all-cause and CV mortality rates among patients with stage 3 or 4 CKD [10]. Furthermore, recent studies have associated lower UA values with higher all-cause and CV mortality among patients on hemodialysis (HD) [11–13]. Therefore, relationships between UA values and all-cause and CV mortality seem quite different depending on whether or not patients undergo dialysis. However, these investigations included relatively small numbers of patients. Therefore, the relationship between UA values and all-cause or CV mortality remains uncertain for patients with CKD, especially those undergoing dialysis. Moreover, whether therapy for hyperuricemia is useful for patients with end-stage kidney disease (ESKD) is also uncertain. The Dialysis Outcomes and Practice Patterns Study (DOPPS) associated higher all-cause or CV mortality with lower UA values, but these findings did not change in a model that included medication with allopurinol [13].

Therefore, we investigated the relationship between UA values and all-cause or CV mortality and the effects of medication for hyperuricemia in patients undergoing HD. We also considered interactions between clinical parameters and all-cause or CV mortality associated with UA or medication for high UA.

## Material and methods

### Design

This observational cohort study investigated associations between UA and all-cause or CV mortality, and the significance of treatment for hyperuricemia in patients on HD.

### Study cohort

The Japanese Society for Dialysis Therapy (JSDT) has conducted annual nationwide surveys of dialysis facilities that address epidemiological background, treatments and the outcomes of dialysis. By the end of 2011, 304,856 patients were undergoing dialysis in Japan [14]. Data were obtained from the standard analysis file, JRDR-13108 with the permission of the Committee of the Renal Data Registry of the Japanese Society for Dialysis Therapy (JRDR). The study protocol was approved by the Medical Ethics Committee of the Japanese Society for Dialysis Therapy and proceeded in accordance with the Declaration of Helsinki (2013). We extracted baseline data from 222,434 patients (age, 67 ± 12 year; male, 63.0%; median dialysis duration, 65 months) who underwent three HD sessions weekly (excluded hemodiafiltration), and whose clinical data including laboratory findings and one-year outcomes were complete. From ethical aspect, all data were fully anonymized before we accessed them.

### Measured parameters

Biochemical parameters including UA, serum albumin, creatinine (Cr), blood urea nitrogen (BUN), total cholesterol, HDL-cholesterol, serum calcium (Ca), serum phosphate, C-reactive protein (CRP) and hemoglobin were measured using standard laboratory techniques at each institution.

Information about all-cause and CV death were extracted from records at the end of 2012. CV death was defined as being caused by heart failure, pulmonary edema, acute myocardial infarction, arrhythmia, endocarditis, valvular disease, subarachnoid hemorrhage, cerebral hemorrhage, cerebral infarction and sudden death.

### Statistical analysis

Data are presented as means ± SD or as medians with interquartile ranges (IQR). Values with P < 0.05 were considered significant. Male and female patients undergoing three HD sessions weekly were compared using Student t-tests, Wilcoxon rank sum tests or chi-square tests. Hazard ratios (HR) and 95% confidence intervals (CI) for all-cause and CV mortality rates among the patients undergoing HD were assessed using Cox regression analysis with the confounding factors of age, gender, HD duration, underlying disease, comorbid disease and laboratory findings (Table 1). A subgroup analyses of specified variables aimed to identify potential interactions affecting risk of mortality. The hazard ratios (HR) and 95% confidence interval (CI) for all-cause, cardiovascular mortality rates were assessed using Cox regression analysis with the confounding factors of all parameters we analyzed listed in Table 1. Data were statistically analyzed using JMP, version 10.0.2 for Windows (SAS Institute Inc., Cary, NC, USA) and STATA version 11.1 (STATA Corporation, College Station, TX, USA).

**Table 1:** Baseline laboratory data and patients’ characteristics.

Variable	All	Male	Female	P*
Number (%)	222434	140150 (63.0%)	82284 (37.0%)	<0.0001
Age (years)	67±12	66±12	68±13	<0.0001
Dialysis duration (months)	65 (28–125)	60 (26–118)	72 (32–138)	<0.0001
Height (cm)	160±10	164±7	151±7	<0.0001
BW (kg)	57.1±12.7	61.4±12.0	49.7±10.4	<0.0001
BMI (kg/m^2^)	22.3±4.3	22.7±4.1	21.8±4.5	<0.0001
Underlying disease				<0.0001
CGN (%)	75974 (34.2%)	45468 (32.4%)	30506 (37.1%)	
Diabetic nephropathy (%)	83736 (37.6%)	57630 (41.1%)	26106 (31.7%)	
Nephrosclerosis (%)	17787 (8.0%)	11519 (8.2%)	6268 (7.6%)	
PKD (%)	7743 (3.5%)	4185 (3.0%)	3558 (4.3%)	
Others or unknown (%)	37192 (16.7%)	21347 (15.2%)	15845 (19.3%)	
Comorbidity				
Hypertension	152808 (68.7%)	98459 (70.3%)	54349 (66.1%)	<0.0001
DM	92901 (41.8%)	63415 (45.2%)	29486 (35.8%)	<0.0001
Acute myocardial infarction	17521 (7.8%)	12803 (9.1%)	4718 (5.7%)	<0.0001
Cerebral hemorrhage	10912 (4.9%)	7095 (5.1%)	3817 (4.6%)	<0.0001
Cerebral infarction	33995 (15.3%)	22696 (16.2%)	11299 (13.7%)	<0.0001
Quadruple amputation	6737 (3.0%)	4915 (3.5%)	1822 (2.2%)	<0.0001
Gouty attack	7267 (3.3%)	5933 (4.3%)	1334 (1.6%)	<0.0001
Laboratory data				
UA (mg/dL)	7.3±1.4	7.3±1.4	7.2±1.4	<0.0001
Alb (g/dL)	3.7±0.4	3.7±0.4	3.6±0.4	<0.0001
BUN (mg/dL)	63±16	63±16	63±16	<0.0001
Cr (mg/dL)	10.1±3.0	10.8±3.1	9.0±2.5	<0.0001
CRP (mg/dL)	0.1 (0–0.4)	0.1 (0–0.4)	0.1 (0–0.3)	<0.0001
Collected calcium (mg/dL)	9.3±0.8	9.2±0.8	9.4±0.9	<0.0001
Phosphorus (mg/dL)	5.2±1.5	5.3±1.5	5.2±1.4	<0.0001
TC (mg/dL)	156±35	149±33	168±36	<0.0001
HDL-C (mg/dL)	48±16	46±15	52±16	<0.0001
Hb (g/dL)	10.5±1.3	10.6±1.3	10.4±1.2	<0.0001
Drugs for hyperuricemia	33988 (15.3%)	24261 (17.3%)	9727 (11.8%)	<0.0001
Allopurinol	31988 (14.4%)	22852 (16.3%)	9136 (11.1%)	<0.0001
Febuxostat	1109 (0.5%)	786 (0.6%)	323 (0.4%)	<0.0001
Others	891 (0.4%)	623 (0.4%)	268 (0.3%)	<0.0001

Data are shown as means ± SD or medians with interquartile ranges (IQR).

*Compared between patients undergoing hemodialysis.

Abbreviations: Alb, albumin; BMI, body mass index; BW, body weight; CGN, chronic glomerular nephritis; Cr, creatinine, DM, diabetes mellitus; Hb, hemoglobin; HDL-C, High-density lipoprotein cholesterol; PKD, polycystic kidney disease; TC, total cholesterol; UA, uric acid.

## Results

Table 1 shows the baseline characteristics of the 222,434 patients included in this study who underwent three HD sessions per week (mean age, 67 ± 12 years; male, 63.0%; median dialysis duration, 65 months). The underlying pathologies comprised chronic glomerulonephritis (CGN) in 75,974 (34.2%), diabetic nephropathy in 83,736 (37.6%), nephrosclerosis in 17,787 (8.0%), polycystic kidney disease (PKD) in 7,743 (3.5%), and others or unknown in 37,192 (16.7%). The numbers of patients with a history of acute myocardial infarction, cerebral bleeding, cerebral infarction, amputation of an extremity, and gout attacks were 7.8%, 4.9%, 15.3%, 3.0% and 3.3%, respectively. Values among males and females for uric acid were 7.3 ± 1.4 and 7.2 ± 1.4 mg/dL, respectively, and 22.7 ± 4.1 and 21.8 ± 4.5, respectively, for BMI. These values were significantly higher in males than in females (P<0.0001). On the other hand, females were significantly older and had been on dialysis significantly longer than males (age, 68 ± 13 vs. 66 ± 12 y; HD duration, 72 (32–138) vs. 60 (26–118) months). Of the 222,434 patients, 18,775 (8.4%) died within one year (male, 11,941 [8.5%]; female, 6,834 [8.3%]), including 8,094 (3.6%) of CV causes (male, 5,004 [3.6%]; female, 3,090 [3.8%]).

**Fig 1:**
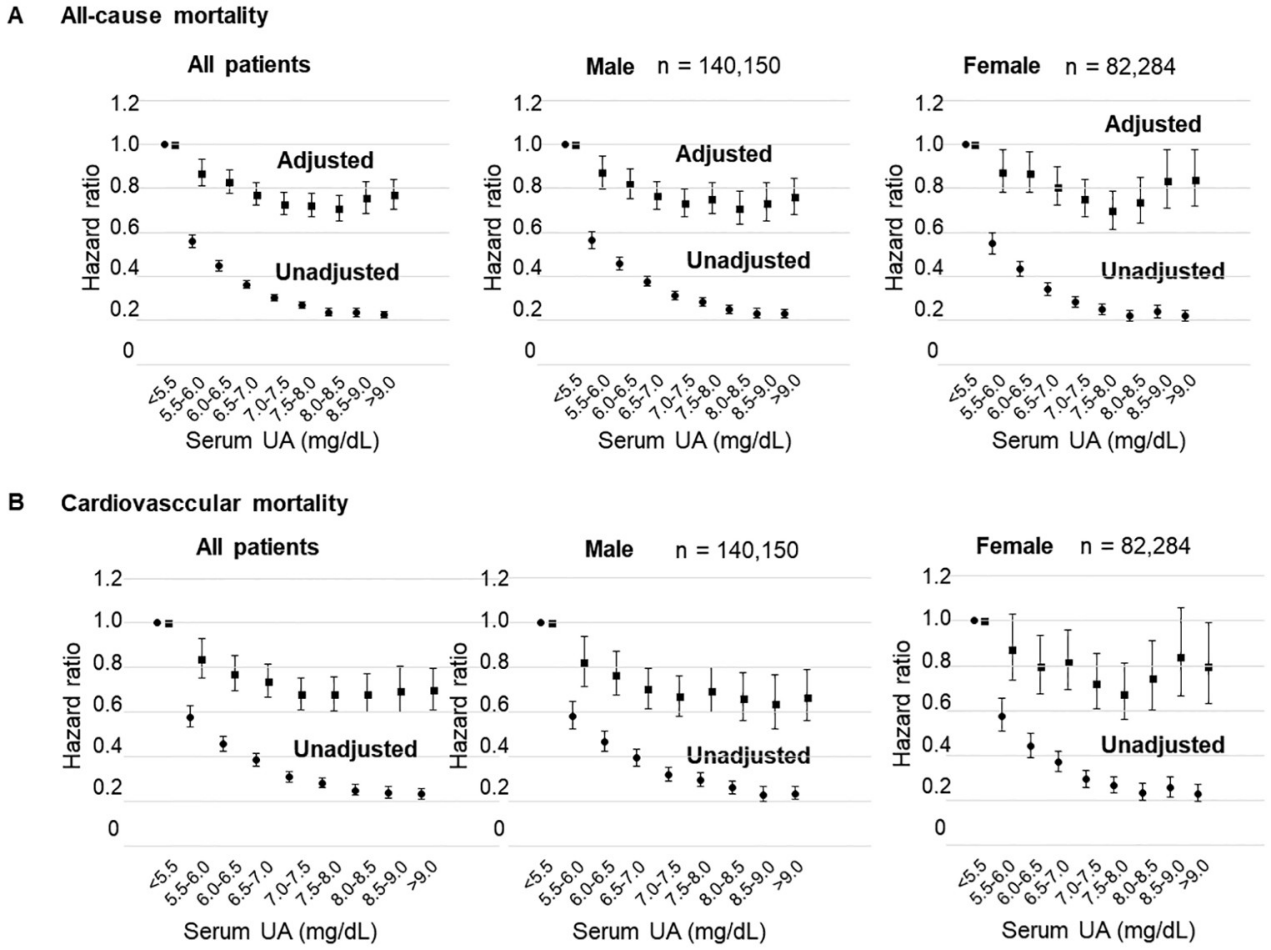
Relationships between serum UA values and one-year mortality. Relationships between serum UA and all-cause or CV mortality (Cox regression analysis) in male, female and all patients. Abbreviations: CV, cardiovascular events; UA, serum uric acid.

**Fig 2:**
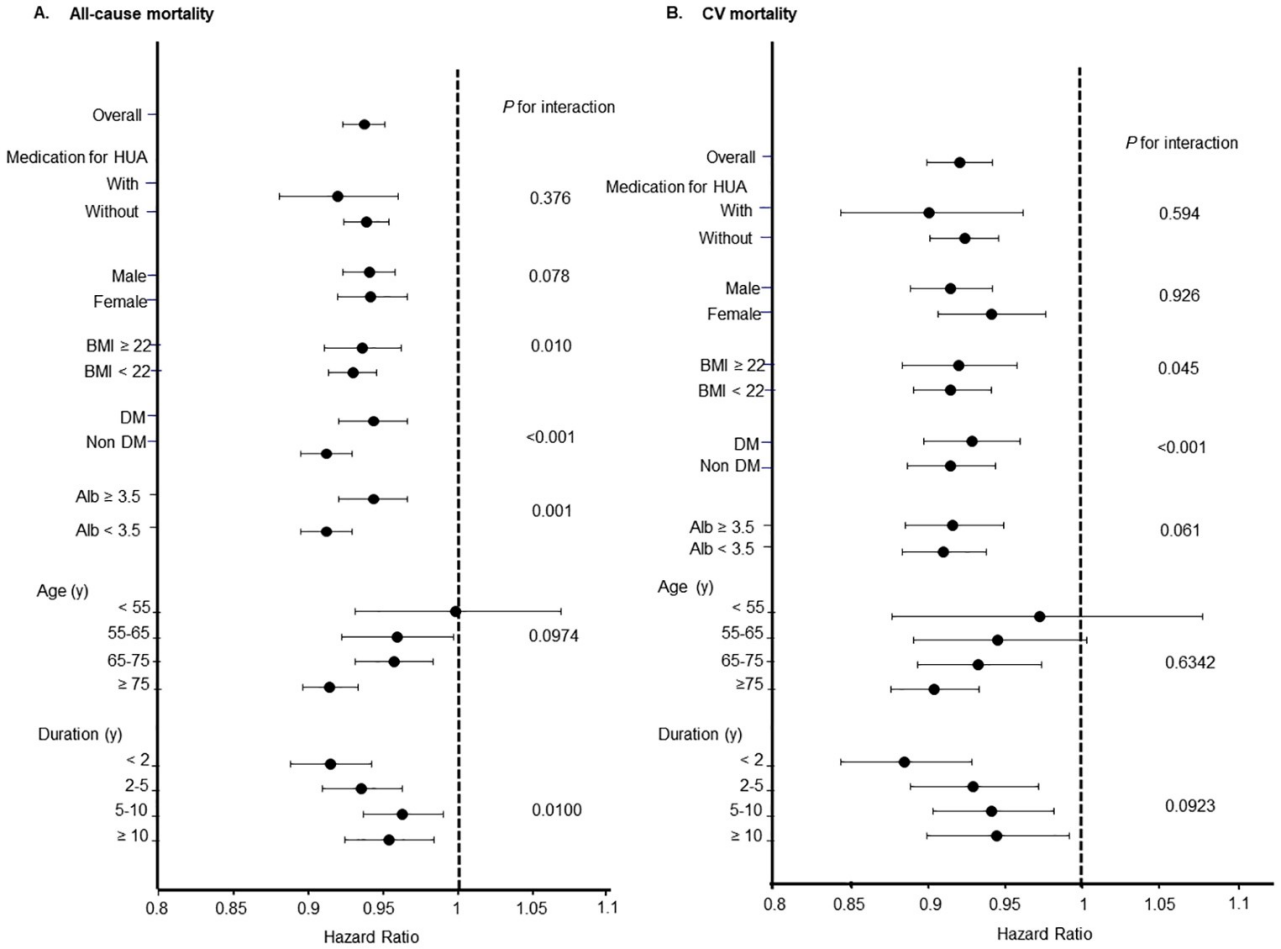
Interactions between effects of serum UA on all-cause or CV mortality and clinical parameters and medication for HUA. A: Effects on interactions affected by serum UA on all-cause mortality. B: Effects on interactions affected by serum UA on CV mortality. Abbreviations: CV, cardiovascular events; HUA, hyperuricemia; UA, serum uric acid.

Tables 2 and 3 shows the HR and 95% CI of all-cause and CV mortality in univariate and multivariate Cox regression analyses. Univariate analysis associated lower UA values with higher all-cause and CV mortality rates. Fig 1 shows that patients with the lowest UA concentrations had the highest all-cause and CV mortality rates, but Fig 2A and 2B shows that male and female interaction was not significant (P for interaction, 0.078 and 0.926, respectively).

**Table 2:** Relationships between serum UA values and all-cause mortality.

All-cause mortality	Unadjusted HR	Adjusted HR
Age (years)	1.067 (1.066 to 1.069)	1.035 (1.033 to 1.038)
Male gender	1.028 (0.998 to 1.059)	1.473 (1.399 to 1.550)
Dialysis duration (months) **	0.989 (0.977 to 1.002)	1.166 (1.141 to 1.192)
BMI (kg/m^2^)	0.858 (0.854 to 0.862)	0.945 (0.938 to 0.952)
DM	1.275 (1.238 to 1.312)	1.255 (1.195 to 1.317)
History of acute MI	1.797 (1.721 to 1.876)	1.342 (1.259 to 1.430)
History of cerebral hemorrhage	2.065 (1.997 to 2.134)	1.122 (1.031 to 1.221)
History of cerebral infarction	2.065 (1.997 to 2.134)	1.201 (1.141 to 1.263)
History of quadruple amputation	2.410 (2.275 to 2.554)	1.367 (1.247 to 1.499)
History of gouty attack	0.709 (0.646 to 0.779)	0.957 (0.842 to 1.089)
UA (mg/dL)	0.714 (0.706 to 0.721)	0.928 (0.911 to 0.946)
Alb (g/dL)	0.175 (0.171 to 0.179)	0.453 (0.428 to 0.481)
BUN (mg/dL)	0.978 (0.977 to 0.979)	1.004 (1.002 to 1.005)
Cr (mg/dL)	0.751 (0.748 to 0.755)	0.848 (0.838 to 0.859)
CRP (mg/dL) **	1.486 (1.473 to 1.499)	1.217 (1.201 to 1.234)
Collected calcium (mg/dL)	1.258 (1.244 to 1.272)	1.091 (1.062 to 1.121)
Phosphorus (mg/dL)	0.775 (0.767 to 0.784)	1.103 (1.084 to 1.122)
TC (mg/dL)	0.992 (0.991 to 0.992)	0.999 (0.998 to 0.9999)
HDL-C (mg/dL)	0.983 (0.982 to 0.985)	0.998 (0.996 to 0.999)
Hb (g/dL)	0.749 (0.741 to 0.757)	0.943 (0.927 to 0.959)
Medication for hyperuricemia	0.591 (0.563 to 0.620)	0.848 (0.786 to 0.916)

Date are shown as means ± SD or medians and interquartile ranges (IQR).

Abbreviations: Alb, albumin; BMI, body mass index; Cr, creatinine; CRP, C reactive protein; DM, diabetes mellitus; Hb, hemoglobin; HDL-C, High-density lipoprotein Cholesterol; MI, myocardial infarction; TC, Total Cholesterol; UA, uric acid.

** Dialysis duration and CRP were log-transformed.

**Table 3:** Relationships between serum UA values and CV mortality.

CV mortality	Unadjusted HR	Adjusted HR
Age (years)	1.063 (1.060 to 1.065)	1.033 (1.029 to 1.037)
Male gender	0.953 (0.911 to 0.997)	1.326 (1.227 to 1.433)
Dialysis duration (months) **	1.003 (0.984 to 1.022)	1.171 (1.132 to 1.211)
BMI (kg/m^2^)	0.873 (0.866 to 0.879)	0.948 (0.937 to 0.958)
DM	1.445 (1.384 to 1.510)	1.458 (1.355 to 1.569)
History of acute MI	2.216 (2.085 to 2.355)	1.631 (1.490 to 1.785)
History of cerebral hemorrhage	2.143 (2.038 to 2.253)	1.079 (0.947 to 1.230)
History of cerebral infarction	2.143 (2.038 to 2.253)	1.278 (1.184 to 1.379)
History of quadruple amputation	2.592 (2.380 to 2.823)	1.459 (1.277 to 1.668)
History of gouty attack	0.673 (0.582 to 0.780)	0.883 (0.721 to 1.082)
UA (mg/dL)	0.719 (0.707 to 0.730)	0.912 (0.886 to 0.939)
Alb (g/dL)	0.207 (0.199 to 0.215)	0.546 (0.498 to 0.597)
BUN (mg/dL)	0.981 (0.979 to 0.982)	1.005 (1.002 to 1.007)
Cr (mg/dL)	0.768 (0.762 to 0.774)	0.853 (0.837 to 0.869)
CRP (mg/dL) **	1.416 (1.398 to 1.434)	1.183 (1.160 to 1.207)
Collected calcium (mg/dL)	1.258 (1.236 to 1.280)	1.132 (1.089 to 1.177)
Phosphorus (mg/dL)	0.817 (0.804 to 0.831)	1.131 (1.102 to 1.161)
TC (mg/dL)	0.993 (0.993 to 0.994)	0.999 (0.998 to 1.0005)
HDL-C (mg/dL)	0.985 (0.983 to 0.986)	0.997 (0.995 to 1.0001)
Hb (g/dL)	0.780 (0.768 to 0.793)	0.948 (0.923 to 0.973)
Medication for hyperuricemia	0.612 (0.569 to 0.658)	0.845 (0.754 to 0.948)

Date are shown as means ± SD or medians and interquartile ranges (IQR).

Abbreviations: Alb, albumin; BMI, body mass index; Cr, creatinine; CRP, C reactive protein; DM, diabetes mellitus; Hb, hemoglobin; HDL-C, High-density lipoprotein Cholesterol; MI, myocardial infarction; TC, Total Cholesterol; UA, uric acid.

** Dialysis duration and CRP were log-transformed.

Fig 2A and 2B shows interactions between the effects of serum UA on all-cause or CV mortality and clinical parameters as well as high UA medication. Interactions were significant between higher mortality rates and lower serum UA, body mass index (BMI), diabetes mellitus (DM), serum albumin and duration of dialysis. Among patients with lower BMI, the absence of DM, lower serum albumin values, a shorter duration of dialysis and a protective effect of higher UA on all-cause mortality were obvious. Similarly, among patients with increased CV mortality and lower UA values, a lower BMI and non-DM become much more obvious. However, lower serum albumin and a shorter duration of HD partially helped to improve CV mortality due to higher UA values.

**Fig 3:**
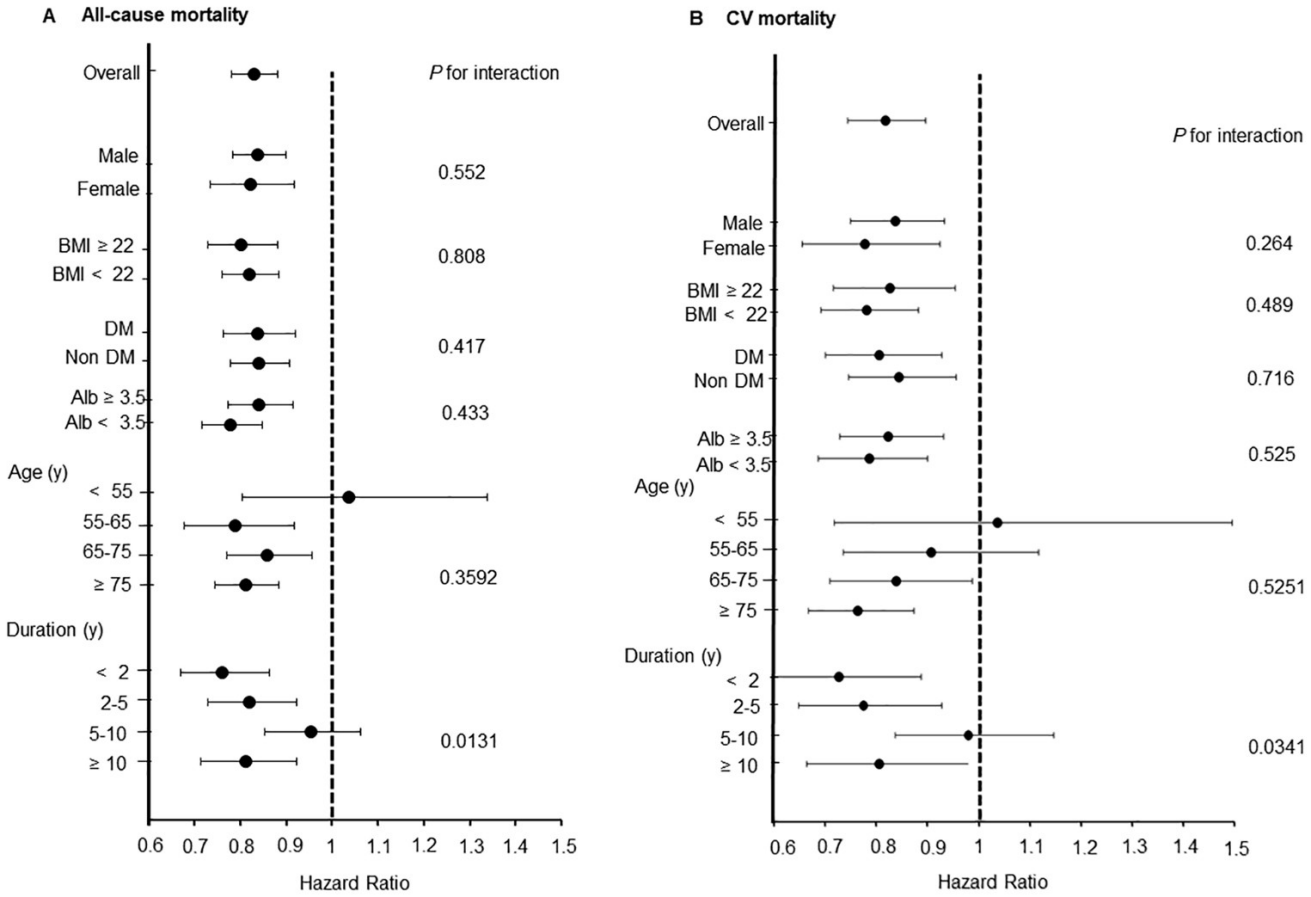
Effects of treatment for hyperuricemia on interactions that influence mortality related to serum UA. A: Effects of treatment for hyperuricemia on interactions that influence all-cause mortality related to serum UA. B: Effects of treatment for hyperuricemia on interactions that influence CV mortality related to serum UA. Abbreviations: CV, cardiovascular events; UA, serum uric acid.

Tables 2 and 3 also shows that medication for hyperuricemia reduced the risk of either all-cause or CV mortality. Fig 3A and 3B shows the results of interactions between the effects of medication for hyperuricemia on serum UA, all-cause or CV mortality, and each clinical parameter. Among patients who had only been undergoing HD for a short period, lower UA values even under medication for hyperuricemia, tended to increase all-cause and CV mortality. There was no interaction of the effects of treatment for hyperuricemia between allopurinol (HR 0.91 (95% CI: 0.87–0.96) of all-cause mortality, HR 0.88 (95% CI: 0.82–0.95) of CV mortality) and febuxostat (HR 0.99 (95% CI: 0.81–1.20) of all-cause mortality, HR 1.02 (95% CI: 0.77–1.34) of CV mortality) (P for interaction, 0.310 and 0.509, respectively). We cannot evaluate about other drugs of treatment for hyperuricemia on interaction, because the number of patients taking other drugs is too small.

## Discussion

We found that lower UA values conferred higher risk for all-cause and CV mortality among patients on HD. Hyperuricemia is a risk factor for developing CKD in normal healthy persons [15]. Patients with reduced renal function have high risk of all-cause and CV mortality [16]. Diabetes mellitus and hypertension frequently cause ESKD, and hyperuricemia is associated with a high incidence of these two conditions [17]. However, the present findings suggested that lower UA is associated with increased risk of all-cause and CV mortality. Even after adjustment for several factors, lower UA remained involved in higher risk of all-cause and CV mortality among males and females. However, Cox regression analyses showed that the effects of UA on mortality were obviously reduced. Uric acid values comprise a surrogate marker of nutritional status, and are associated with inflammation and muscle status [12]. Moreover, the results of stratified analyses for serum albumin, also a surrogate marker of inflammation, were similar to those before stratification. Beberashvili et al. [18], and the DOPPS study [13] also found lower UA values in patients on HD; therefore the present results support the previous findings. These studies also noted that lower UA suggests malnutrition and low protein intake. Mortality rates are high for patients on ESKD who also have malnutrition-inflammation-atherosclerosis (MIA) syndrome [19]. Therefore, the present findings suggest that lower UA values represent lower nutritional status and increased mortality. Moreover, the present study included far more patients than previous studies, therefore the data are highly credible. However, some residual confounding factors of nutrition might exist.

The interactive factors of higher mortality due to lower UA values are BMI and DM. High BMI and DM are associated with many life-threatening complications such as atherosclerosis, CV events and increased susceptibility to infection. Therefore, these could also be confounding factors. Serum albumin values and dialysis duration are also interactive factors of all-cause mortality due to lower UA. Serum albumin is a surrogate marker of inflammation, and thus, low serum albumin concentrations imply an inflammatory state. These conditions might be fatal, and thus possible confounding factors of higher all-cause mortality rates due to lower UA. Moreover, a longer period of dialysis will result in more complications. However, some patients who have been on HD for a very long time might have started HD when quite young. Such patients at the time of HD initiation probably had very few complications, which might explain a lower mortality rate, whereas cardiovascular events are associated with advanced age. Thus, the duration of HD might not be an interactive factor in CV mortality.

Medication for hyperuricemia has profound significance for mortality. The survival advantage for patients under medication for hyperuricemia is decreased. Since UA is a surrogate marker of nutritional status, if UA remains high under medication, then hyperuricemia is uncontrollable because the nutritional status is as high as that among healthy persons, and it seems to increase the risk of mortality. Moreover, many studies have suggested that high UA is a risk factor for the onset of new CKD. Although Kuriyama et al. [20] suggested that high UA is associated with new hypertension, but not with the incidence of CKD during a follow up period of four years, a recent review by Tsai et al., suggested that high UA is associated with the development of new CKD [15]. These results suggest that high UA leads to CKD over the long term. A uricase inhibitor to administered to a rat model of hyperuricemia did not increase blood pressure [21]. The xanthine oxidase (XO) inhibitor, allopurinol, not only lowers UA but reportedly reduces the progression of renal disease [22].

The mechanism of renal protection might not depend on blood pressure. A previous study has suggested that patients on HD have decreased plasma levels of antioxidants, and that XO activity is an independent predictor of CV events in such patients, regardless of UA [23]. Therefore, other factors, such as oxidative stress, are associated with the progression of renal insufficiency in hyperuricemia. One mechanism of a renal protective effect is via the suppression of oxidative stress; therefore, XO inhibitors might reduce oxidative stress. However, whether XO inhibitors suppress oxidative stress remains controversial. Clinical and experimental animal studies have found that febuxostat suppresses oxidative stress [24–27], whereas allopurinol does not reduce oxidative stress in patients with diabetes [28]. The present study associated lower UA values with all-cause and CV mortality rates even under medication for hyperuricemia. However, the range of HR was wider in the group that was administered with medication for hyperuricemia than in a group that was not. This means that lower UA under medication for hyperuricemia indicates malnutrition, but also therapeutic success. Medication for hyperuricemia has profound significance for improving mortality; therefore, the results (Fig 2A and 2B) do not mean that mortality rates are elevated because of urate-lowering therapy. Low UA indicates high mortality, but an adequate UA level due to therapy for hyperuricemia ameliorates mortality as a consequence. However, we cannot suggest an adequate UA target value for medicines that treat hyperuricemia. Moreover, we did not investigate oxidative stress. However, even if patients with ESKD receive medication for hyperuricemia and have suppressed oxidative stress, high oxidative stress is likely to be generated because of CKD and hyperuricemia even when UA values remain uncontrollable. Therefore, the significance of medication for hyperuricemia is important for protection against oxidative stress and reducing the risk of mortality. Moreover, the sole interactive factor in the effects of medication for hyperuricemia on all-cause and CV mortality and serum UA level is the duration of dialysis. This might be the same as the relationships between lower UA values and all-cause and CV mortality. Patients with a longer duration of HD might have many complications associated with mortality, and medical intervention for high UA cannot improve mortality. Therefore, medication for hyperuricemia might be more effective against mortality among patients with a shorter duration of HD.

The present study has several limitations. We did not have a control group, and the observational design allowed only limited conclusions. Therefore, we cannot prove a cause-and-effect relationship between UA values and mortality. We measured UA and other laboratory parameters only at baseline, and could not determine the effects of changes from baseline during follow-up using time-dependent analyses. The period when data were collected preceded the widespread availability of febuxostat or topiroxostat, and thus we cannot discuss their effects on patients undergoing HD.

In conclusion, although a lower serum UA concentration is associated with increased risk of all-cause and CV mortality in patients on HD, treatment for hyperuricemia might improve their outcomes. However, multivariate analysis showed that the relationship between lower UA and mortality was suppressed, UA might not be an independent risk factor for death and several confounding factors might exist. Therefore, the significance of intervention for hyperuricemia among patients on HD warrants further discussion.
